# Structural coalescence underlies the aggregation propensity of a β-barrel protein motif

**DOI:** 10.1371/journal.pone.0170607

**Published:** 2017-02-10

**Authors:** Carla R. Angelani, Julio J. Caramelo, Lucrecia M. Curto, José M. Delfino

**Affiliations:** 1 Universidad de Buenos Aires, Facultad de Farmacia y Bioquímica, Departamento de Química Biológica, Buenos Aires, Argentina; 2 Universidad de Buenos Aires, Consejo Nacional de Investigaciones Científicas y Técnicas, Instituto de Química y Fisicoquímica Biológicas (IQUIFIB), Facultad de Farmacia y Bioquímica, Universidad de Buenos Aires, Buenos Aires, Argentina; 3 Universidad de Buenos Aires, Facultad de Ciencias Exactas y Naturales, Departamento de Química Biológica, Buenos Aires, Argentina; 4 Universidad de Buenos Aires, Consejo Nacional de Investigaciones Científicas y Técnicas, Instituto de Investigaciones Bioquímicas de Buenos Aires (IIBBA), Facultad de Ciencias Exactas y Naturales, Universidad de Buenos Aires, Buenos Aires, Argentina; 5 Fundación Instituto Leloir, Buenos Aires, Argentina; Kermanshah University of Medical Sciences, ISLAMIC REPUBLIC OF IRAN

## Abstract

A clear understanding of the structural foundations underlying protein aggregation is an elusive goal of central biomedical importance. A step toward this aim is exemplified by the β-barrel motif represented by the intestinal fatty acid binding protein (IFABP) and two abridged all-β sheet forms (Δ98Δ and Δ78Δ). At odds with the established notion that a perturbation of the native fold should necessarily favor a buildup of intermediate forms with an enhanced tendency to aggregate, the intrinsic stability (ΔG°_H2O_) of these proteins does not bear a straightforward correlation with their trifluoroethanol (TFE)-induced aggregation propensity. In view of this fact, we found it more insightful to delve into the connection between structure and stability under sub-aggregating conditions (10% TFE). In the absence of the co-solvent, the abridged variants display a common native-like region decorated with a disordered C-terminal stretch. Upon TFE addition, an increase in secondary structure content is observed, assimilating them to the parent protein. In this sense, TFE perturbs a common native like region while exerting a global compaction effect. Importantly, in all cases, fatty acid binding function is preserved. Interestingly, energetic as well as structural diversity in aqueous solution evolves into a common conformational ensemble more akin in stability. These facts reconcile apparent paradoxical findings related to stability and rates of aggregation. This scenario likely mimics the accrual of aggregation-prone species in the population, an early critical event for the development of fibrillation.

## Introduction

Achieving full understanding of the mechanism of protein aggregation will represent a breakthrough in the context of both physiological and pathological phenomena occurring in nature. Such information will likely be of great use to shed light on normal processes or-when the outcome goes astray- on the origin of pathologies. Undoubtedly, this new comprehension will be of fundamental value in establishing modes of intervention on aggregation diseases with effector molecules, hopefully leading to the development of new therapies [1,2].

Natural β-sheet structures present different mechanisms to avoid edge-to-edge mediated aggregation. Particularly, β-barrel motifs escape this situation, because they tend not to expose free edges by establishing a continuous β-hydrogen bonding network organized all around the barrel [3]. There are few proteins of the β-class which are useful as model systems for protein engineering, mainly due to their conspicuous tendency to aggregate. For this reason, the intestinal fatty acid binding protein (IFABP) family arises as a very helpful target that allows molecular intervention on those structural determinants underlying conformational change, folding, misfolding and aggregation.

**Fig 1:**
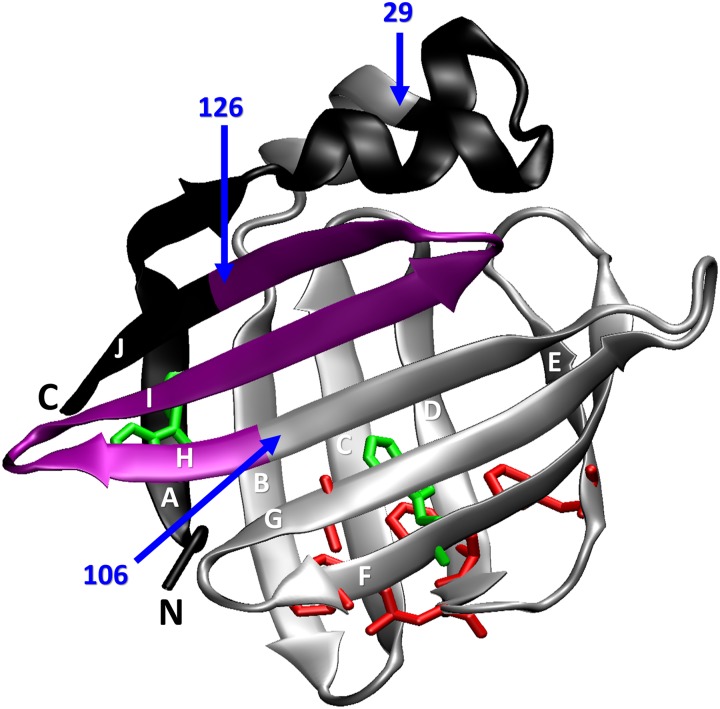
Ribbon structure of IFABP (PDB 2IFB). For Δ98Δ, the excised N- and C-terminal fragments are shown in black. The Δ78Δ variant -lacking also the 107–126 fragment (in purple)-adopts a dimeric structure. Residues belonging to the hydrophobic core (F47, F62, L64, F68, M84 and L89) are depicted with their side-chains in red polytube representation. W82, that also belongs to the hydrophobic core, and W6 are shown in green polytubes. The figure was generated with VMD [4] and rendered with POV-Ray.

From a structural standpoint, FABPs are monomeric antiparallel β-barrel proteins consisting of two five-stranded β-sheets (named βA-βE and βF-βJ, Fig 1). These sheets are arranged in a nearly orthogonal orientation enclosing the ligand binding cavity. The FABP barrel is flattened and displays an ample discontinuity between βD and βE. All β-strands are connected by β-turns with the exception of βA and βB, where an intervening helix-turn-helix motif appears. The latter is formed by two short but well-defined α-helices, which pack onto the top of the fatty acid-binding site. At variance with most globular proteins, the inner space of FABPs is occupied by a large solvent-filled cavity, whereas the small hydrophobic core is displaced from the protein center.

To improve our knowledge on key structural and functional determinants of this protein family we devised an approach involving the systematic abbreviation of IFABP, thus generating two functional variants. To this end, we took advantage of the ‘selection’ imposed by controlled proteolysis to deconstruct the β-barrel. The first abridged variant: Δ98Δ (Fig 1, [5,6]) is a stable, monomeric and functional form of IFABP, including only the 98 amino acid residues corresponding to the sequence 29–126 of IFABP. By comparison with the full-length protein, Δ98Δ is devoid of βA, most of the helical domain and the last five amino acids belonging to βJ. This truncation leads to the loss of both stretches involved in the closure of the β-barrel. Despite this fact, Δ98Δ retains substantial β-sheet content and native-like tertiary interactions. Most significantly, all the critical residues of the hydrophobic core involved in the nucleation event leading to the folded state are conserved. Δ98Δ retains the ability to bind fatty acids and might fold through an analogous hierarchical scheme as that described for IFABP. Similarly, Δ78Δ was obtained after proteolysis of Δ98Δ [7]. This new fragment lacks an additional C-terminal stretch (107–126). Here again, all the residues belonging to the hydrophobic core are preserved. Diverse spectroscopic techniques show that Δ78Δ adopts a stable well-folded state. The most distinctive feature of Δ78Δ is that it adopts a dimeric structure capable of binding fatty acids. In hindsight, one could rationalize that preservation of the hydrophobic core arises as an essential demand to attain proper folding and function.

As has already been proven, perturbation around the core region leads to amyloid-like aggregation [8,9]. In this scenario, this protein family emerges as a useful model system to explore critical determinants leading to β-aggregates. At odds with the common notion that a perturbation of the native fold should necessarily favor the population of aggregation-prone species, we found that even though these proteins share a common amyloidogenic stretch, their intrinsic stability (ΔG°_H2O_: IFABP≥Δ78Δ>Δ98Δ) does not bear a straightforward correlation with their aggregation propensity triggered by 25% v/v 2,2,2-trifluoroethanol (TFE, Δ78Δ>IFABP>Δ98Δ, [8,9]. In this context, it might be more insightful to correlate the aggregation tendency with the stability measured in the presence of this co-solvent. For this reason, we put our focus on characterizing the changes in conformation and stability of these proteins upon the addition of a sub-aggregating concentration of TFE. Ultimately, our aim here was to contribute to shed light on the early stages of the mechanism leading to fibrillation.

## Materials and methods

### Materials

IFABP, Δ98Δ and Δ78Δ were expressed and purified as described previously [6,7,10]. 2,2,2-trifluoroethanol (TFE), 1-anilino naphthalene-8-sulfonic acid (ANS), trans-parinaric acid (tPA), oleic acid, urea and buffers were purchased from Sigma-Aldrich (St. Louis, MO). Protein concentration was estimated by ultraviolet (UV) absorption: ε_280nm_ = 15910, 9154 and 6970 M^-1^ cm^-1^ for IFABP, Δ98Δ and Δ78Δ respectively.

### Circular dichroism

Spectra were recorded on a Jasco J-810 spectropolarimeter. Ellipticity data in the near UV (250–320 nm) or in the far UV (200–250 nm) regions were collected at 25°C using cuvettes of 10 or 1 mm path length, respectively. A scan speed of 20 nm min^-1^ with a time constant of 1 s was used. Each spectrum was measured three times and the data was corrected for the background signal of buffer and averaged to minimize noise. Molar ellipticity was calculated as described elsewhere [11], using mean residue weight values of 114.4, 112.0 or 111.5 for IFABP, Δ98Δ or Δ78Δ, respectively. The CD spectra of the proteins (10 μM) incubated with oleic acid or trans-parinaric acid (protein to fatty acid ratio of 1:4 or 1:1, respectively) were measured in both the far and near UV regions.

### Fluorescence measurements

Fluorescence measurements were performed at 25°C in an Aminco Bowman Series 2 or a Jasco FP-6500 spectrofluorimeter equipped with a thermostated cell. A 1 cm or a 3 mm path cuvettes sealed with Teflon caps were used. Proteins were dissolved in buffer PN8 (5 mM sodium phosphates buffer, 150 mM NaCl, pH 8.0) in the absence or in the presence of the indicated concentrations of TFE. For intrinsic fluorescence measurements, protein concentration was 15–20 μM. Excitation wavelength was 295 nm and the emission was collected in the range 310–410 nm. The spectral slit-widths were set to 3 nm for each monochromator. For the ANS binding assay, the protein concentration was 2 μM and the ligand was added from a stock solution to a final concentration of 25 μM. Incubation for 3 min ensured complete equilibration. The excitation wavelength was set to 400 nm and emission spectra were collected in the range 420–600 nm, the spectral slit-widths for each monochromator were 4 and 8 nm, respectively. Displacement of bound ANS by oleic acid was estimated from the decrease of fluorescence intensity upon the addition of oleic acid (10 μM final concentration). When necessary, data were corrected for dilution and inner filter effects [12]. For each spectrum, the total integrated intensity and the maximum wavelength of fluorescence emission (λ_max_) were the parameters used for further analysis.

### Fluorescence quenching

Quenching by acrylamide of the intrinsic fluorescence of proteins was investigated at 25°C by sequentially adding aliquots (30 μL) of a stock acrylamide solution (4 M) to a solution of each protein (12 μM) dissolved in buffer PN8 (2 mL) in the absence or in the presence of TFE (10% v/v). Quenching data were analyzed according to the Stern-Volmer formalism:

$$\frac{F_{o}}{F}=1+K_{SV}\left[Q\right]$$

where F_0_ and F are the integrated emission intensities in the absence or in the presence of the quencher Q, respectively, and K_sv_ is the Stern-Volmer constant.

### Size-exclusion chromatography (SEC-FPLC)

Protein solutions (100 μL) were sampled onto a Superdex-75 column (GE Healthcare Amersham Biosciences) equilibrated in PN8 in the absence or in the presence of TFE (10%, v/v). Protein samples were prepared in each buffer and centrifuged (16100 x g at room temperature) before injection. The flow rate was set to 0.3 or 0.2 mL min^-1^ for buffer alone or buffer added with TFE (10%, v/v), respectively. Elution profiles were recorded following the UV absorption at 280 nm, and the multi-angle static light scattering (MASLS) and dynamic light scattering (DLS) signals from in-line modules (Wyatt Technology). Data processing was carried out with the ASTRA software (Wyatt).

### Limited proteolysis

Limited proteolysis experiments were carried out by incubating IFABP, Δ98Δ or Δ78Δ with clostripain (Arg C), chymotrypsin or proteinase K. All enzymatic digestions were performed in PN8 at 30°C in the absence or in the presence of TFE (10% v/v) using enzyme to substrate ratios of 1:20 or 1:200 (w/w). The progress of the reaction was monitored by sampling the incubation mixture at different time intervals.

Proteolytic fragments were analysed by ESI MS after in-line reverse phase HPLC separation on a C4 column (Vydac). ESI ion trap MS analyses were carried out in a ThermoFisher LCQ-Duo with ion trap detector installed at the LANAIS-PROEM facility (UBA-CONICET, Buenos Aires).

### Thermal denaturation

Thermal unfolding was monitored by the change of the dichroic signal at 216 nm. Each protein sample (200 μl, 12 μM) contained in a 1 mm cell cuvette was gradually heated from 25 to 95°C at a scan rate of 0.5°C min^-1^. For proteins that denature reversibly according to a simple two-state transition, involving the unfolded state (U) in equilibrium with the native structure (N), the following equations [13] were fitted to the data:

$$\Delta G_{NU}=-RT\text{ln}\left(\frac{f_{U}}{f_{N}}\right)=\Delta H_{Tm}+\Delta C_{P}\left(T-Tm\right)-T\left(\left(\frac{\Delta H_{Tm}}{Tm}\right)+\Delta C_{P}\text{ln}\left(\frac{T}{Tm}\right)\right)$$

$$S=f_{N}\left(S_{o,N}+l_{N}T\right)+f_{U}\left(S_{o,U}+l_{U}T\right)$$

where *f*_*N*_ and *f*_*U*_ *(= 1-f*_*N*_*)* are the folded (native) and unfolded fractions at equilibrium, respectively, and *T*_*m*_ is the temperature at which *f*_*U*_ equals *f*_*N*_. At any given point, the observed CD signal (*S*) is interpreted as the sum of the signals from the native (*S*_*N*_) and unfolded (*S*_*U*_) forms present in the equilibrium mixture. Assuming a linear dependence of *S*_*N*_ and *S*_*U*_ with temperature, *S*_*0*,*N*_ and *S*_*0*,*U*_ are the intrinsic CD signals for the native and unfolded states, respectively, and *l*_*N*_ and *l*_*U*_ are the slopes of the pre and post transition regions, respectively.

For experiments run in the presence of 10% v/v TFE, the existence of a concomitant process involving thermally-induced aggregation prevents the application of this model. In such cases, the temperature of the onset of aggregation (T_*o*_) was measured instead, as shown in S9 Fig. Briefly, this value is derived after fitting a straight line to the initial time points, and extrapolating backwards to intersect the temperature abscissa.

### Chemical denaturation

Conformational transitions were monitored as a function of denaturant concentration by measuring the change in the intrinsic fluorescence intensity of the proteins. Individual samples of protein (12 μM final concentration) ranging in denaturant concentration from 0 to 8 M urea were prepared by dilution of a fixed volume of a stock solution of protein in mixtures of PN8 and 9 M Urea. Samples were analyzed after incubation for at least 1 h to ensure that the equilibrium had been reached. Non-linear least-squares fits to the equilibrium data were achieved using an equation representing a two-state model for protein denaturation, adapted from Bolen and Santoro [14]. For the dimeric construct Δ78Δ, a minimal two-state model considering simultaneous dissociation and unfolding (D ↔ 2U) was used for the fitting [7].

## Results

**Fig 2:**
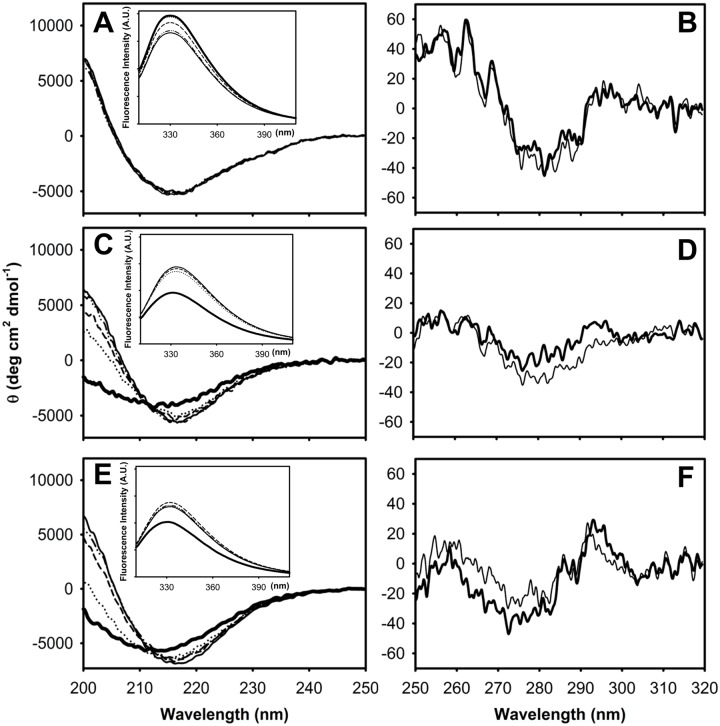
Circular dichroism spectroscopy of IFABP (A, B), Δ98Δ (C, D) and Δ78Δ (E, F). Far (left panels) and near (right panels) UV CD spectra at increasing TFE concentrations (% v/v): 0 (solid thick line), 2.5 (dotted line), 5 (dashed line), 7.5 (dash-dot-dot-dashed line) and 10 (solid thin line). Fluorescence spectra corresponding to the same samples are shown as insets. The ordinate axes, indicating Fluorescence intensity arbitrary units are drawn in the same scale.

The co-solvent TFE is generally employed to induce helical structures on peptides, but it can also perturb the native state of proteins [15]. Therefore, the effect of low concentration of TFE on the conformation of IFABP and the abridged variants Δ98Δ and Δ78Δ was assayed. For IFABP, no change is observed -either in the far or in the near UV CD spectra- a fact indicative of lack of any significant conformational change (Fig 2A and 2B). However, a different behavior is observed for the abridged variants (Fig 2C–2E). Even at the lowest concentration assayed (2.5%), each abbreviated protein suffers a dramatic change in the shape of the far UV CD spectrum. The minimum is red-shifted (4 nm), giving rise to a spectrum coincident with that observed for the parent protein: similar molar ellipticity and minimum at ~ 216 nm. Additionally, a strong positive band centered at ~ 200 nm appears. Above that concentration, the spectra do not further change in shape, but the latter signal progressively increases in magnitude. Remarkably, at 10% TFE the far UV CD spectrum of all three proteins is almost identical. In the near UV CD range, minor spectral changes take place by the addition of 10% TFE, indicating a full conservation of the fine structure.

IFABP contains two tryptophan residues: W82, which is buried within the hydrophobic core and W6, placed at the N-terminal β-strand (strand A, Fig 1). The only remaining such residue in the variants (W82) is known to contribute approximately 75% of the fluorescence emission and there is almost no cross-talk between both tryptophan residues in the parent protein [16]. Thus, W82 becomes a useful spectroscopic probe to evaluate the integrity of the hydrophobic core. For IFABP, upon the addition of TFE (up to 10%), fluorescence emission intensity decreases, whereas the position of the maximum wavelength of emission is conserved (λ_max_ 330 nm, inset to Fig 2A). It should be noted that the expected effect of TFE on the fluorophore -as revealed by experiments run on N-acetyl-L-tryptophanamide (NATA)- is a blue shift of the emission peak and a decrease in quantum yield [17]. Conversely, for Δ98Δ and Δ78Δ -even at the lowest TFE concentration assayed (2.5%)- there is a large increase in the intensity and a 2 nm red shift in λ_max_ (331 to 333 nm) (inset to Fig 2C and 2E). Collectively, these results can be interpreted as a phenomenon of conformational coalescence of all three proteins. Importantly, all TFE-induced changes proved to be reversible (S1 Fig).

**Fig 3:**
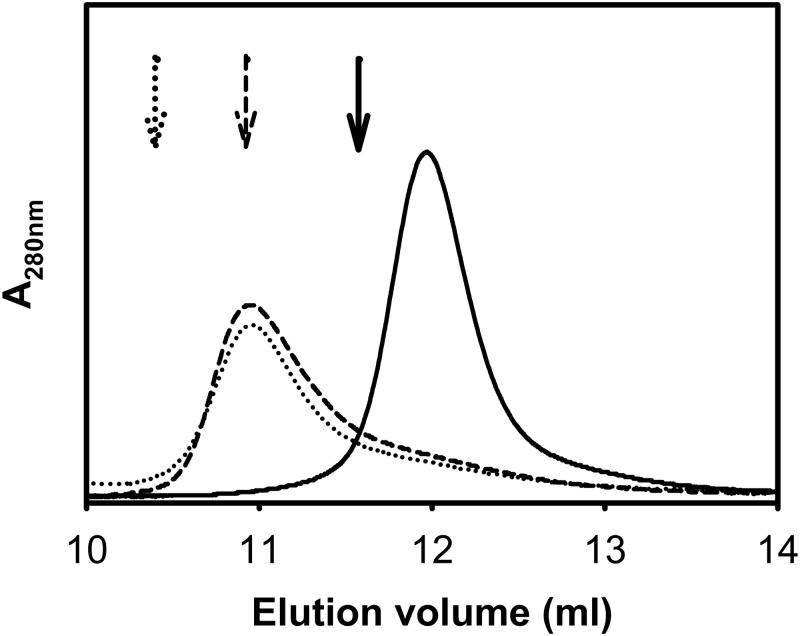
Size-exclusion chromatography. Size-exclusion chromatography of IFABP (solid line), Δ98Δ (dashed line) and Δ78Δ (dotted line). Proteins were sampled onto a Superdex-75 column and eluted at 10% v/v TFE in buffer PN8 (see Materials and methods). Arrows indicate the elution volumes in the absence of TFE.

To explore putative changes triggered by TFE in the hydrodynamic properties of the proteins we studied their behavior by size exclusion chromatography (Fig 3). In the absence of TFE the Stokes radius of the abridged variants is known to be larger than that predicted for a globular protein of the same molecular weight [5,7]. Static light-scattering evidence confirms that no change in the oligomeric state of the proteins occurs up to 10% TFE (S2 Fig). In addition, the hydrodynamic radius of full-length IFABP and dimeric Δ78Δ is lower in the presence of TFE, pointing to this co-solvent exerting a compaction or sphering effect on these two proteins.

**Fig 4:**
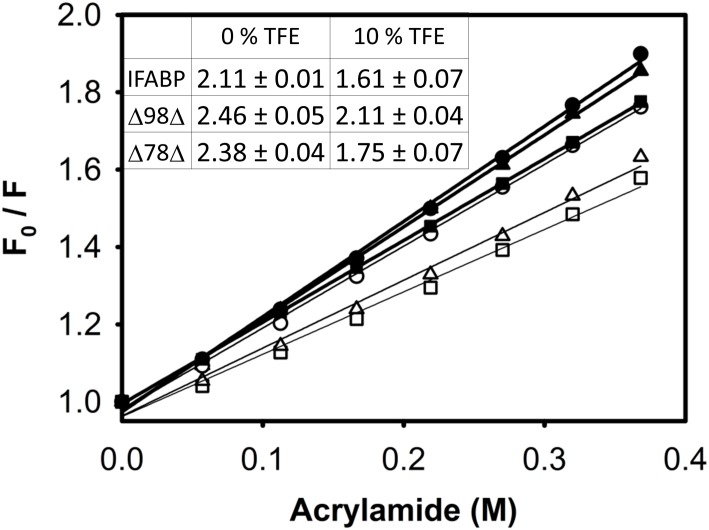
Fluorescence quenching. Quenching by acrylamide of the intrinsic fluorescence intensity of IFABP (■, □), Δ98Δ (●,○) and Δ78Δ (▲,△). Experiments were carried out in the absence (closed symbols) or in the presence of 10% v/v TFE (open symbols) in buffer PN8 (see Materials and methods). The values of the Stern-Volmer constants are shown as an inset table.

To evaluate the solvent accessibility of the protein core, quenching by the neutral molecule acrylamide was investigated (Fig 4). Stern-Volmer constants (Ksv) are lower in the presence of TFE, revealing a diminished solvent exposure of W82. These results go in line with the compaction effect observed in Fig 3. Here again, the addition of the co-solvent exerts a stronger effect on Δ78Δ and IFABP than on Δ98Δ: lower Ksv values amounting to decreases of approx. 26 and 24% for the former, as compared to 14% for the latter. A similar control experiment using NATA showed identical Ksv values at 0 and 10% TFE, ruling out any trivial effect of the co-solvent on indole fluorescence (data not shown).

**Fig 5:**
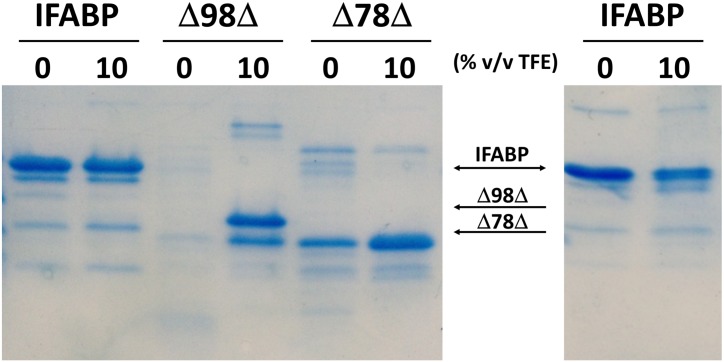
SDS-PAGE separation. Separation by SDS-PAGE of the digestion mixture of proteins after treatment with chymotrypsin. Proteins were digested at a mass ratio of protein to protease of 200:1 for 30 min at 30°C (left panel) and 20:1, overnight at 30°C (right panel).

To study the impact of TFE on the flexibility of these proteins, limited proteolysis experiments were carried out (Fig 5). At 10% TFE, the abridged variants are more resistant to degradation by chymotrypsin, pointing to a decrease in conformational flexibility. Similar results are obtained with proteinase K (S3 Fig). This evidence is consistent with the observed consolidation of the secondary structure, as revealed by CD (Fig 1C–1F). It has been reported that the addition of up to 15% TFE causes slight changes in the secondary structure of chymotrypsin, thus reducing the rate of proteolysis [18]. Nevertheless, under similar experimental conditions (10% TFE), IFABP does not show any difference in the digestion pattern. Given this fact, the protein was challenged with the protease under severe conditions (right panel in Fig 5). Indeed, a lower amount of remaining intact protein is observed for the sample incubated at 10% TFE. This behavior is similar to that observed for other native well-folded proteins such as α-lactalbumin, bovine serum albumin or lysozyme (S4 Fig), i.e. they become more easily degraded in the presence of TFE.

**Fig 6:**
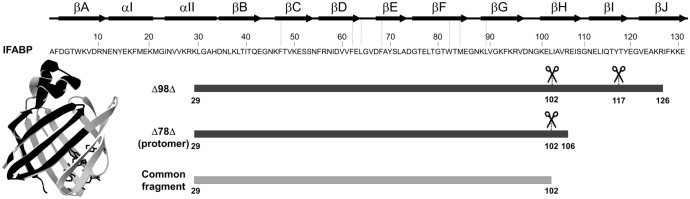
Amino acid sequence and schematic representation of the secondary structure elements of IFABP. Amino acid residues belonging to the hydrophobic core are indicated with dotted lines and depicted with their side-chains in poly-tube representation on the ribbon structure of IFABP (PDB 2IFB). Linear depiction of the abridged variants and of a common early proteolytic fragment (below in light gray) obtained after cleavage by chymotrypsin. The latter is also painted in light gray in the 3D model.

Additional evidence on the effect of TFE on the conformation of the proteins can be obtained from the analysis of the early proteolytic events. Variants Δ98Δ and Δ78Δ were digested at a mass ratio of protein to chymotrypsin of 200:1 at 30°C, either in the presence or in the absence of TFE. Samples were taken at different time intervals and subjected to MS-spectrometry analysis. Since the monomeric variant Δ98Δ is rapidly digested, samples were taken at shorter times of proteolysis than for Δ78Δ. Globally, the available data points to the presence of a peptide comprising the stretch 29–102 of IFABP (8366.9 Da) as the common proteolysis product (Fig 6 and S5 Fig). Significantly, it preserves all the amino acid residues belonging to the hydrophobic core. Δ98Δ (11089.6 Da) gives rise to fragment 11055.7 Da (29–117) as the main component in mixture with peptide 29–102. At a slower rate, this same pattern is observed in 10% TFE. In addition, Δ78Δ directly brings forth peptide 29–102. However, in 10% TFE a substantial amount of intact protomer (8807.0 Da) remains in the mixture. The quantitative differences in the rate of proteolysis observed for the abridged variants might be attributed to a co-solvent induced decrease in conformational mobility.

**Fig 7:**
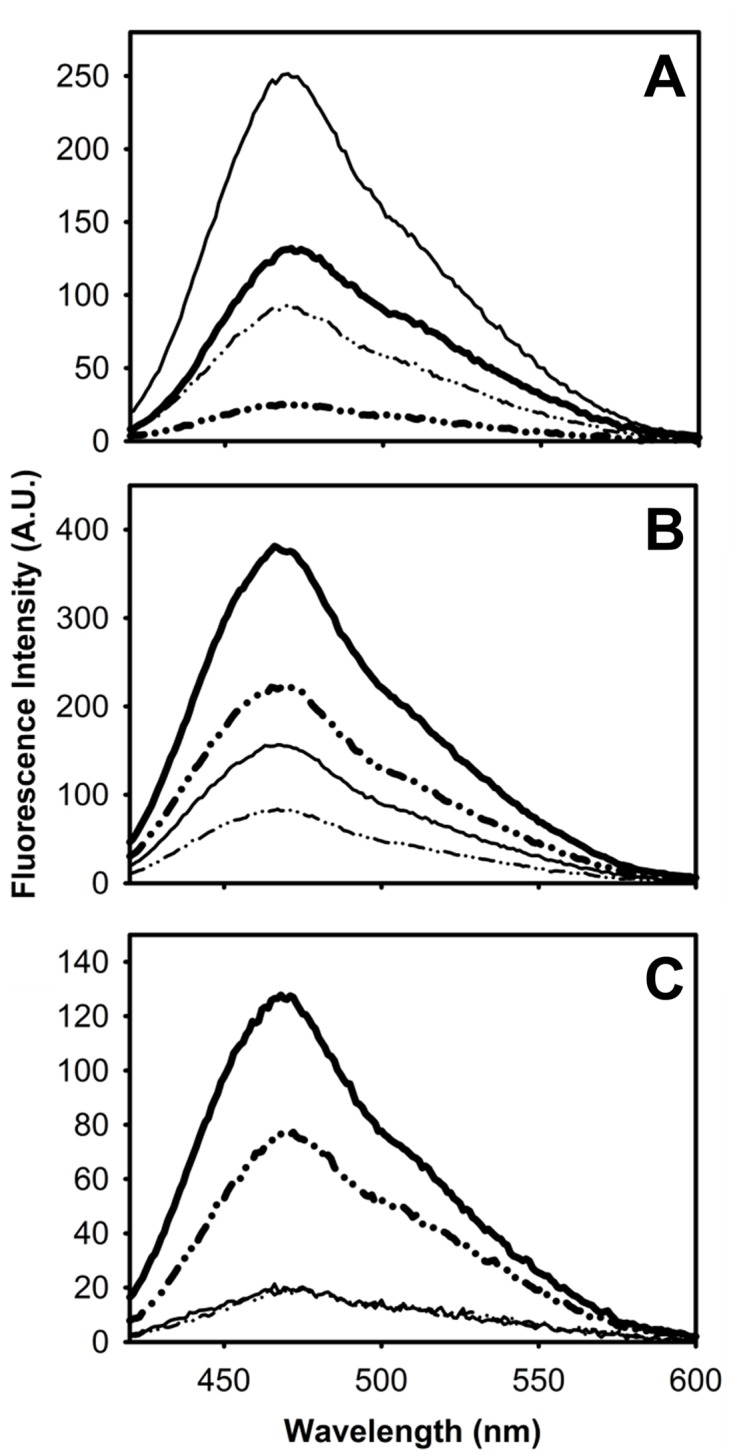
ANS binding. Binding of ANS to IFABP (A), Δ98Δ (B) and Δ78Δ (C). The intensity of fluorescence emission of ANS was measured when bound to proteins at 0 (thick lines) or 10 (thin lines) % v/v TFE in buffer PN8 (see Materials and methods). Spectra obtained in the presence of oleic acid at a 5:1 molar ratio with respect to protein are represented in dash-dot-dot-dash lines.

Unlike other proteins in the native state either FABP or its variants are able to bind he fluorescent probe 1-anilino naphthalene-8-sulfonic acid (ANS) within the ligand binding cavity [6,7,10]. However, they differ in their displacement behavior with the natural ligand oleic acid (18:1). The nature and binding affinity of the observed sites in all three proteins have been extensively characterized in previous publications of our group [6,7,10]. This picture provides the background against which the effect of the TFE co-solvent on the exposure of hydrophobic areas is analyzed (Fig 7). When no TFE is present, ANS bound to the parent protein is completely displaced by this fatty acid (8% remaining fluorescence), while in the abridged variants a sizeable amount of ANS remains bound, even in the presence of a large excess of fatty acid. This observation reveals the existence of a binding site that is absent in the full-length protein.

When IFABP is pre-incubated in 10% TFE, close to a two-fold enhancement of the ANS fluorescence emission is observed. Conversely, for both variants, the presence of TFE leads to a dramatic decrease in the intensity (~60% or ~80% for Δ98Δ or Δ78Δ, respectively). Next, the addition of an excess amount of oleic acid was assayed. Samples with no protein but with the same amount of this fatty acid and the probe ANS show negligible fluorescence (results not shown). In the presence of TFE, the remaining fluorescence of ANS bound to IFABP accounts for 44% of the original signal, indicating the appearance of a new non-displaceable binding site. Under the same condition, the non-displaceable site is still observed for Δ98Δ, whereas Δ78Δ is no longer able to bind ANS. Consequently, for the latter the addition of oleic acid does not further change the emission spectrum.

Direct evidence of fatty acid binding to IFABP and its variants in the presence of the TFE co-solvent comes from two experimental approaches using CD spectroscopy: (i) the first evaluates conformational changes occurring as a consequence of oleic acid binding (S6 Fig), and (ii) the second measures the appearance of induced bands of the ligand trans-parinaric acid (t-PA) when lodged into the asymmetric protein environment (S7 Fig). Previous reports from our laboratory pointed to a ligand-induced ordering effect exerted by oleic acid on the abridged variants, but not on full-length IFABP [6,7,10].

In the presence of TFE only slight changes become evident as oleic acid binds to these constructs, a behavior reminiscent of that observed for IFABP. Importantly, in all cases the same final spectra are obtained regardless of the order of addition of components to the mixture (oleic acid or TFE), pointing to a clear equilibrium situation.

A similar experiment was carried out using the polyunsaturated fatty acid t-PA (18:4). It has been shown that the affinity of IFABP and Δ98Δ for t-PA is higher than that measured for oleic acid (Kd = 0.13 and 0.72 μM, respectively, [5]). Interestingly, Δ78Δ retains the ability to bind this ligand (Kd = 0.4 μM), displaying lower affinity than IFABP, but higher than Δ98Δ [7]. By itself, this fatty acid does not show any intrinsic optical activity, thus enabling its use as a probe to study protein binding sites by CD. When t-PA becomes bound, the protein scaffold provides an asymmetric environment that brings about the appearance of new dichroic bands. Changes in the binding site involving the immediate t-PA milieu also support the existence of a structure-inducing effect promoted by the co-solvent TFE on all three proteins. Particularly, the use of this probe led us to obtain direct biophysical evidence on the ability of Δ78Δ to bind fatty acids in the presence of TFE.

**Fig 8:**
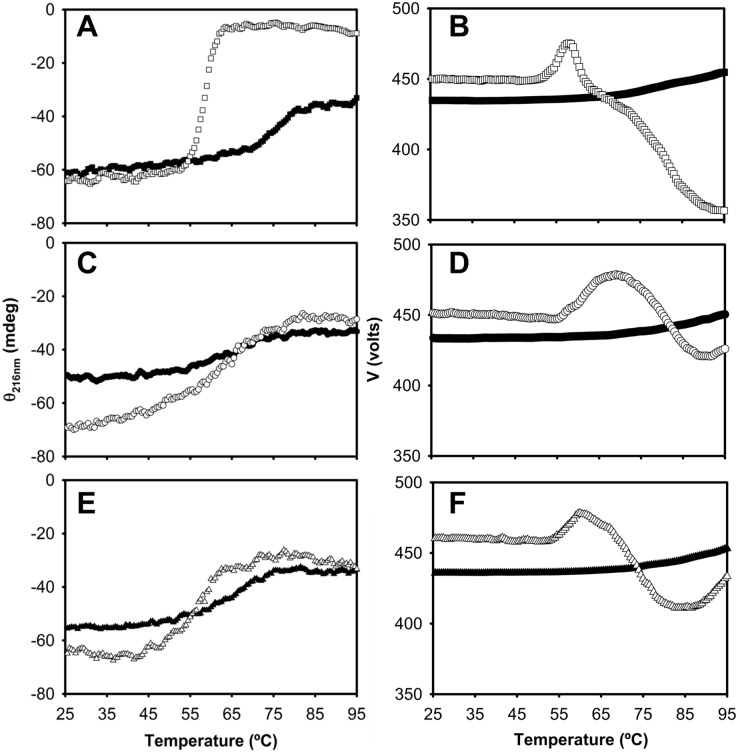
Thermally-induced unfolding transitions. Circular dichroism (CD) measurements recorded as temperature is increased for IFABP (A and B), Δ98Δ (C and D) and Δ78Δ (E and F). The transitions were monitored by the evolution of the ellipticity signal at 216 nm (left panels) at 0 (closed symbols) and 10% v/v TFE (open symbols). The value of the dynode voltage (V in Volts) at each condition is shown (right panels).

To understand the global impact of TFE on the stability of these proteins, we proceeded to evaluate their temperature and urea-induced denaturation profiles. A model for a reversible two-state transition could be fitted to the thermal data obtained in the absence of TFE (Fig 8). The values of the midpoint temperature of denaturation (Tm) thus measured were 76, 64 and 67°C for IFABP, Δ98Δ and Δ78Δ, respectively. In contrast, this model failed to be fitted to the data acquired in the presence of 10% TFE. Under this condition, an increase in the dynode voltage (voltage applied to the photomultiplier tube to compensate for the reduction in the light intensity) reveals the presence of turbidity due to the appearance of protein aggregates that evolve into a precipitate (see Fig 8B, 8D and 8F). Since turbidity causing light scattering can be recorded simultaneously along the collection of CD data, this approach allows a direct correlation between protein conformational changes and aggregation behavior [19]. As both processes occur concomitantly, the transitions are inherently irreversible and cannot be analyzed by a formalism assuming thermodynamic equilibrium. For IFABP full-blown aggregation is observed, whereas both abridged variants exhibit this phenomenon to a much lesser extent. Indeed, the cooling CD data shown in S8 Fig reveals a virtually null trace for IFABP, indicative of a massive loss of protein into clumps. Conversely, Δ98Δ and Δ78Δ display a cooperative cooling behavior compatible with a partially reversible process. In this scenario, one cannot properly define Tm, therefore we will refer to the temperature of the onset of aggregation (T*o*), as defined in S9 Fig. Remarkably, in the presence of the co-solvent TFE, T*o* values become very similar for the three proteins assayed: 52, 57 and 54°C for IFABP, Δ98Δ and Δ78Δ, respectively. In summary, the largest drop is observed for the parent protein, whereas the abridged variants exhibit a lesser effect.

**Fig 9:**
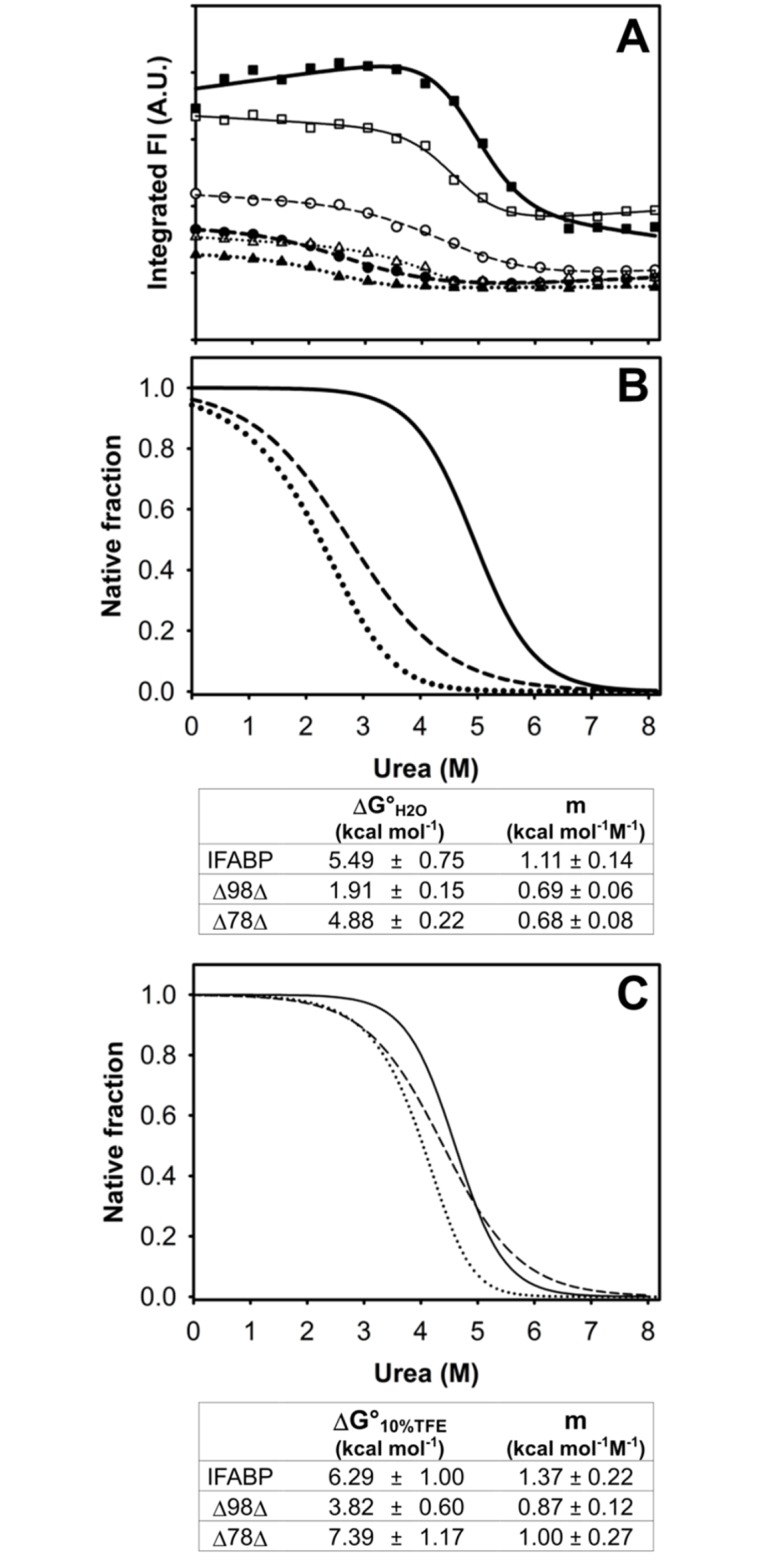
Urea-induced unfolding transitions. Evolution of the fluorescence emission (integrated intensity) as a function of urea concentration (Panel A) for IFABP (■, □), Δ98Δ (●,○) and Δ78Δ (▲,△). Experiments were carried out in the absence (closed symbols) or in the presence of 10% v/v TFE (open symbols). Fitted curves of the native molar fraction of IFABP (solid line), Δ98Δ (dashed line) or Δ78Δ (dotted line) are plotted at 0 (panel B) and 10% v/v TFE (panel C). Notice that for the dimeric construct Δ78Δ -that has been described to dissociate and unfold concomitantly- the free energies (ΔG° values) of the overall process (expressed in protomer equivalents, see reference [7]) are tabulated.

Subsequently, urea-induced equilibrium unfolding denaturation experiments were carried out (Fig 9). As has been previously reported, in the absence of TFE, both abridged variants show a cooperative transition behavior [5,7]. Their stability was characterized by the free energy of unfolding (ΔG°_H2O_) in the following order: IFABP>Δ78Δ>Δ98Δ. Remarkably, in the presence of the co-solvent, the stability of both abridged variants increases significantly, whereas that for the full-length protein remains unchanged. Although in the presence of TFE the slopes of the fitted curves appear to be slightly higher, the changes observed are not significant. Strikingly, in the latter condition, the transition midpoints are very similar for the wild-type and abridged proteins (~ 4.6, 4.4 and 4.0 M for IFABP, Δ98Δ and Δ78Δ, respectively). In summary, the structure-promoting effect of TFE leads to a merger of the unfolding transitions curves.

## Discussion

Previous work from of our laboratory [8,9] showed that the aggregation of IFABP, Δ98Δ and Δ78Δ triggered by 25% TFE share a common nucleation-elongation mechanism. The first event is a fast equilibrium step, whereby TFE induces a conformational rearrangement of the native protein (P), giving rise to an aggregation-prone state (P*). In all cases, the ensuing process includes the formation of a dimeric nucleus followed by the growth of the fibrillar aggregates by the irreversible apposition of more monomer [8,20]. Contrary to common expectation, the intrinsic stability of these proteins in water does not bear a straightforward correlation with their aggregation propensity. This observation is at odds with the established notion that a perturbation of the native fold would necessarily favor the population of aggregation-prone species [21]. In this sense, it might be more insightful to correlate aggregation propensity with conformational stability measured in the presence of up to 10% TFE, the maximal concentration at which all proteins remain soluble.

A main effect of TFE, as supported by the far UV CD data, is a general increase of secondary structure content for both partially disordered forms (Fig 2). Interestingly, the co-solvent exerts this effect–in band intensity and position of the wavelength of the minimum- at very low concentrations up to a limit coincident with that shown by the full-length protein.

Nevertheless, as no 3D structure is available for either of the variants, only theoretical approaches can shed light on the localization of stretches involved in this TFE-induced conformational change [22–25]. Predictors of disorder point to regions comprising around the initial 15 and the last 30 amino acid residues of the full-length protein as the least ordered zones. Moreover, the presence of three molecular recognition features (MoRFs, [26]) in the 8–13, 60–68 and 118–131 regions were also found. According to predictions, the middle stretch coincides with the main amyloidogenic segment [9]. It is likely that potentially disorder-prone tails of IFABP can play at least two different functions. First, they might be involved in the closure of the β-barrel, and second, they might serve as ‘entropic bristles’ [27] preventing IFABP from oligomerization along the hierarchical process [28] proposed for the folding of this protein [9]. Remarkably, a major part of the predicted disordered C-terminal tail is preserved in Δ98Δ, whereas it is virtually absent in Δ78Δ. For Δ98Δ, it can be speculated that the C-terminal remainder might act as an entropic bristle inhibiting aggregation. By contrast, in the case of Δ78Δ, due to its much shorter length, it would be unable to exert that role, thus the construct will adopt a stable dimeric form [9]. Accordingly, preliminary data derived from coarse-grain molecular dynamics simulations indicates that the C-terminal segment in any of the abridged variants would be the last region attaining the final fold (work in progress).

Information derived from proteolysis (Figs 5 and 6) indicates that both variants are digested to a lesser extent in the presence of TFE, suggesting that this co-solvent might reduce the flexibility of the C-terminal stretch up to, at least, residue 102 (this segment might even be longer since the next predicted proteolytic site would occur at F93). Due to its proximity to the compact hydrophobic core, this position might lie next to a more ordered region. In the case of the abridged variants, the effect exerted by TFE might in part implicate helical induction at the otherwise disordered C-terminal section. This putative gain in α-helical structure would imply a decreased flexibility for this zone, thus conceivably explaining the observed changes in the far UV CD spectra and their lower digestion propensity. Indeed, both J-Pred [29] and Yaspin [30] algorithms predict an intrinsic propensity of the segment 122–128 of IFABP to adopt α-helical conformation (S10 Fig). For Δ78Δ, even though its C-terminal stretch is shorter, a similar situation might also be taking place.

The current available data allows us to refine the structural picture postulated for the abridged proteins: these might display a common native-like region decorated with a disordered C-terminal stretch. Suggestively, all proteolysis products conserve the N-terminal end. It is known that the effects of TFE upon secondary structural elements in the framework of a protein might differ from those observed with small peptides [31]. In this sense, the co-solvent might exert a dual function, by perturbing the native-like region [15] and concurrently inducing helical structure along the disordered C-terminal segment. As a global structural consequence of the co-solvent addition, a significant compaction effect is also at work. This phenomenon attains significant extent for Δ78Δ, it is of a rather lower magnitude in the case of IFABP and becomes negligible for Δ98Δ (Fig 3). Solvent accessibility to the core in the presence of TFE is modified accordingly (Fig 4). On the other hand, IFABP constitutes a scaffold less prone to disorder. As judged by CD and intrinsic fluorescence spectroscopies (Fig 1A and 1B), the parent protein suffers only very minor structural alterations in the presence of 10% TFE. Despite this fact, the increased ANS fluorescence (Fig 7) and its enhanced sensitivity to proteolysis (Fig 5) show that TFE exerts deleterious structural perturbations. This picture is at variance with that observed for both abridged variants. Here, increased secondary structure and more defined tertiary signatures become evident. In addition, the impact of TFE on the core region is somewhat greater than that for IFABP, as attested by an increase in the intrinsic fluorescence intensity and a 2 nm red shift in λ_max_ (Fig 1C–1F). The decrease of both the propensity to proteolysis (Fig 5) and the exposure of hydrophobic areas (Fig 7) goes in line with the proposed structure-promoting effect of TFE.

Cumulative evidence discussed above supports the notion that the structural rearrangements brought about by 10% TFE lead to the conformational coalescence of all three proteins, a process resulting also in a trio more akin in stability (Figs 8 and 9). Interestingly, the dimeric variant appears to be no longer able to bind ANS in the presence of TFE. This can be rationalized by a shrinking of the hydrophobic pocket and/or by a sterically hindered access to the binding cavity for a bulky and rigid ligand such as ANS. Most significantly, even under the influence of TFE, the proteins remain functional as they are still able to bind fatty acids (S6 and S7 Figs). Importantly, one should recall that the solvent filled cavity in FABP is much larger than the volume occupied by a fatty acid [32]. As retaining this functionality is signature of the native state, it can be concluded that this level of co-solvent favors the population of alternative conformations scarcely explored in water. Additionally, as these proteins share a common aggregation-prone fragment, variations in the rate of this process might be rationalized in terms of differences in protein conformation, that in turn will determine the exposure/accessibility of this critical stretch [9]. In all likelihood, the TFE-induced sub-states are more akin in structure and stability than the collection of sub-states in water. Even though structural coalescence does not suffice by itself to establish a straightforward correlation between stability and aggregation propensity, it goes a step forward along this way. Indeed, in the presence of TFE IFABP becomes less tightly packed and is much more susceptible to thermal unfolding. Conversely, under this same condition the structural consolidation of the abridged variants gives rise to forms with higher stability. This effect is especially manifest for the monomeric variant Δ98Δ. Suggestively, it has been reported that an increase in helical content reduces aggregation propensity [21], a fact that might help explain why this variant shows the lowest aggregation propensity. For Δ78Δ, it was proposed that the higher rate of aggregation lies in its dimeric nature, a fact that simplifies an otherwise bimolecular nucleation process into a unimolecular one [9]. In this scenario, a low concentration of TFE might foster conformational changes akin to those leading to aggregation-prone species. This experimental approach helps understand early conformational changes controlling the onset of aggregation.

## Supporting information

S1 Fig Circular dichroism (CD) spectra of IFABP (A), Δ98Δ (B) and Δ78Δ (C). Far UV CD spectra are shown at 0 (thick line) and 10 (thin line) % v/v TFE before (solid line) and after (dashed line) dialysis of the samples. Near UV CD spectra corresponding to 0 and 10% v/v TFE after dialysis are plotted as insets.
(TIF)

S2 Fig SEC-FPLC of the abridged variants. Profiles of Δ98Δ (A, C) and Δ78Δ (B, D) in the absence (upper paneles) or in the presence of 10% TFE (lower panels). The right ordinate axes correspond to molecular weight data estimated by multi-angle static light scattering (MASLS). Average values (and standard deviations in Da) are the following: A: 12400 (1600); B: 16000 (900); C: 16700 (3700); D: 13700 (1600).
(TIF)

S3 Fig Proteolysis analysis using proteinase K. Separation by SDS-PAGE of the digestion mixture after treatment of proteins with proteinase K at 0 and 10% v/v TFE. Proteins were digested at a mass ratio of protein to protease of 200:1 for 30 min at 30°C.
(TIF)

S4 Fig Proteolysis analysis using chymotrypsin. SDS-PAGE analysis of the fragments obtained by limited proteolysis with chymotrypsin at mass ratio of protein to protease of 10:1, overnight at 30°C. The lane labeled C corresponds to a control of protein load onto the gel.
(TIF)

S5 Fig ESI MS spectra of proteolysis products of the abridged variants. Proteins were digested at a mass ratio of protein to chymotrypsin of 200:1 at 30°C, either in the absence (upper paneles) or in the presence of 10% TFE (lower panels). The analysis of mixtures arising from Δ98Δ at 5 min (A) or 12 min (C) and those from Δ78Δ at 30 min (B and D) are shown in each panel.
(TIF)

S6 Fig Oleic acid binding. Oleic acid binding to IFABP (A and B), Δ98Δ (C and D) and Δ78Δ (E and F). Far (left panels) and near (right panels) UV CD spectra are shown at 0 (thick line) and 10 (thin line) % v/v TFE in buffer PN8 (see Materials and methods). Spectra recorded in the presence of oleic acid are represented in red lines. Smoothing of the traces (B, D and F) was achieved by averaging twice on a 15-point moving window (1.5 nm). The signature of oleic acid binding in the far UV CD region is a change of shape of the spectrum, characterized by a deepening and a trend toward incremental positive ellipticity at 200 nm (panels C and E). In the presence of 10% TFE, upon ligand binding a further deepening of the minimum and broadening in the range 215–235 nm are observed for all proteins. All in all, the effect of the co-solvent assimilates the response of the abridged variants to that observed for the parent protein. This evidence falls in place within a picture describing a general ordering effect on the truncated constructs. Consistently, the ligand-inducing effect appears as a significant enhancement of the magnitude of the near UV CD spectral signals observed for the abridged variants (panels D and F).
(TIF)

S7 Fig trans-parinaric acid binding. Binding of trans-parinaric acid (t-PA) to IFABP (A and B), Δ98Δ (C and D) and Δ78Δ (E and F). Far (left panels) and near (right panels) UV CD spectra are shown at 0 (thick line) and 10 (thin line) % v/v TFE in buffer PN8 (see Materials and methods). Spectra recorded in the presence of the fatty acid are represented in red lines. Smoothing of the traces (B, D and F) was achieved by averaging twice on a 15-point moving window (1.5 nm). For the abridged proteins in buffer, the new dichroic bands are: a negative one centered at ~ 247 nm and a positive band centered at ~ 235 nm (panels C-F). Notice the common iso-dichroic point at ~ 240 nm. For comparison, two very weak bands of opposite sign also appear in the case of IFABP. The more rigid framework provided by the wild-type protein places the ligand in a more asymmetric location, as evidenced by the appearance of a fine-structured CD spectrum in the near UV region (panels A and B). TFE leads to several perturbations in the spectra: (i) the intensity of the band centered at ~ 235 nm becomes higher for IFABP and Δ78Δ, whereas a red shift occurs for Δ98Δ; (ii) for all proteins, the band initially centered at ~ 247 nm suffers a dramatic red shift (~ 15 nm). For IFABP and Δ78Δ, a substantial enhancement of the intensity is also observed.
(TIF)

S8 Fig Circular dichroism analysis. CD measurements recorded upon cooling samples of IFABP (A), Δ98Δ (B) and Δ78Δ (C) from 95 to 25°C. The temperature transitions were monitored by the evolution of the ellipticity at 216 nm at 0 (closed symbols), and 10 (open symbols) % v/v TFE.
(TIF)

S9 Fig Calculation of the temperature of the onset of aggregation (T*o*). This value is derived after fitting a straight line to the initial time points, and extrapolating backwards to intersect the temperature abscissa. The monochromator was set at 216 nm. The voltage applied to the photomultiplier tube (PMT) of the spectropolarimeter is represented on the Y-axis, as the difference (ΔV) with respect to the value measured at 25°C.
(TIF)

S10 Fig Secondary structure propensity for the C-terminal segment of IFABP (93–131) as predicted by the algorithms JPred and Yaspin. E and H stand for extended and helical conformations. Dashes correspond to positions where no defined secondary structure can be predicted or loops in the experimental structure. The latter was determined by X-ray crystallography (PDB 2IFB).
(TIF)

## Abbreviations

Δ78Δ: a truncated variant of IFABP corresponding to the fragment 29–106 of the parent protein
Δ98Δ: a truncated variant of IFABP corresponding to the fragment 29–126 of the parent protein
λ_max_: position of the maximum wavelength of fluorescence emission
IFABP: intestinal fatty acid binding protein
TFE: 2,2,2-trifluoroethanol
