# Supplementary figures and images for: Structural coalescence underlies the aggregation propensity of a β-barrel protein motif
Source: PLoS One. 2017 Feb 10;12(2):e0170607. doi: 10.1371/journal.pone.0170607 (PMC5302452; doi:10.1371/journal.pone.0170607)

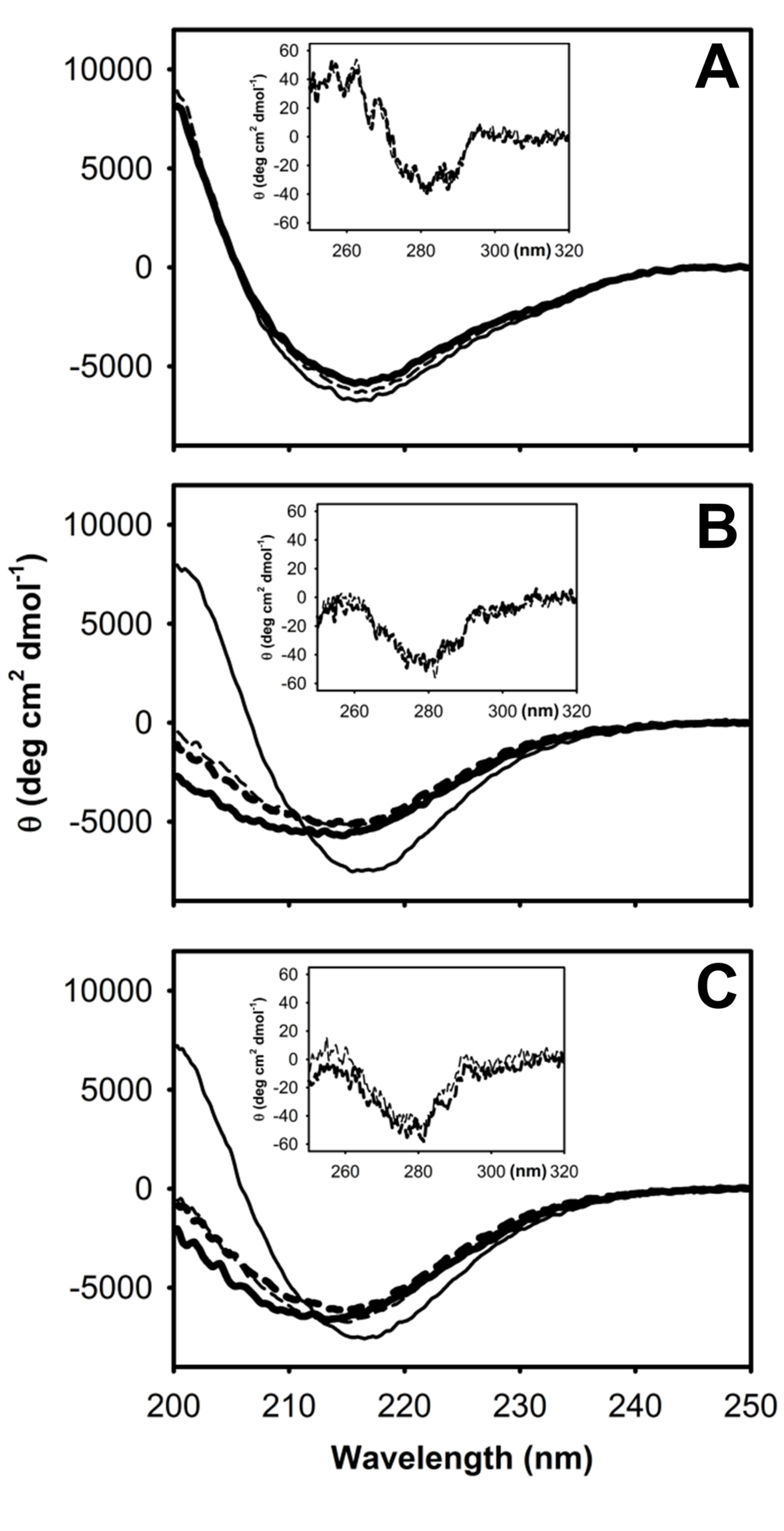

Supplement: S1 Fig — Far UV CD spectra are shown at 0 (thick line) and 10 (thin line) % v/v TFE before (solid line) and after (dashed line) dialysis of the samples. Near UV CD spectra corresponding to 0 and 10% v/v TFE after dialysis are plotted as insets. (TIF) [file pone.0170607.s001.tif]

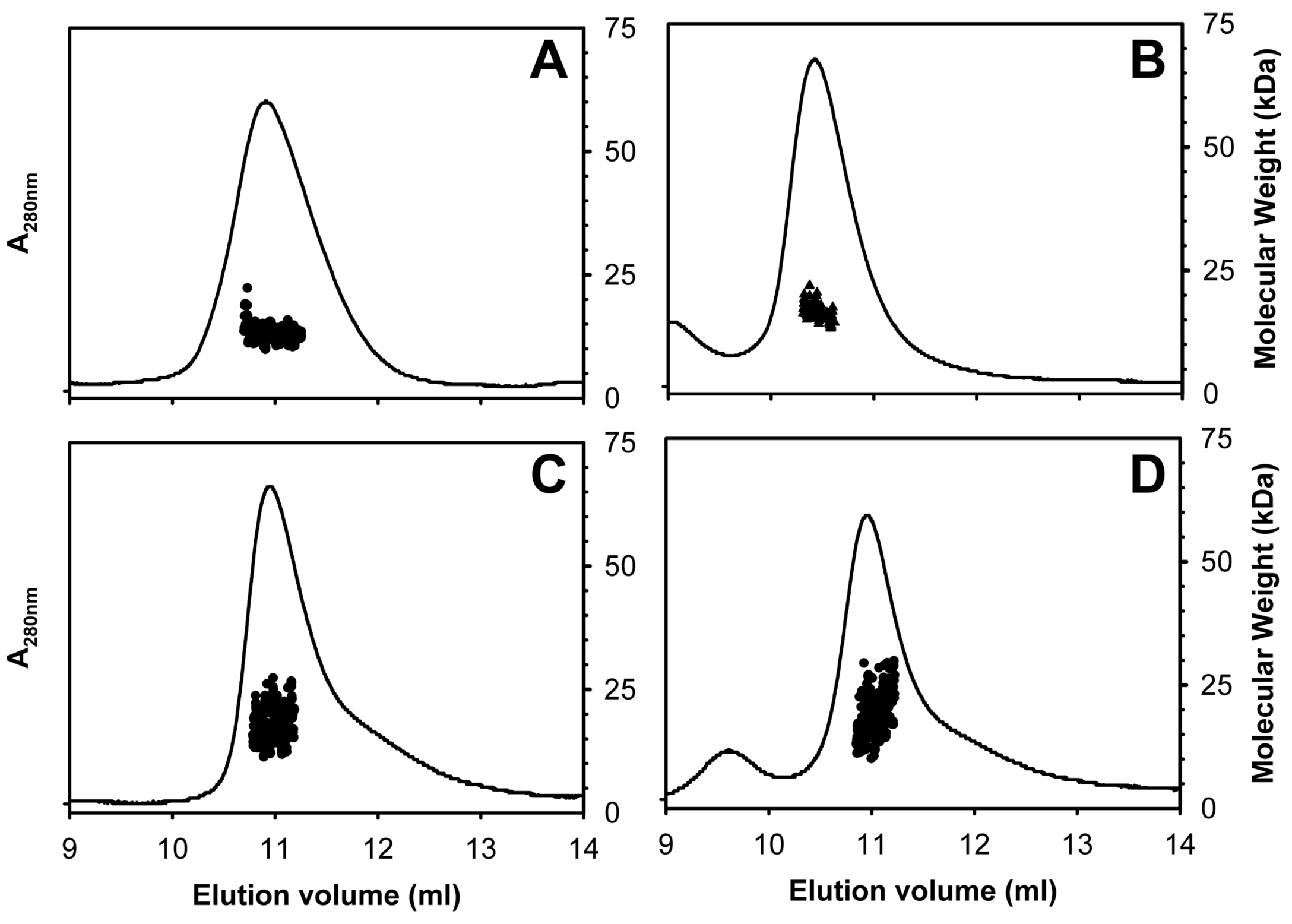

Supplement: S2 Fig — Profiles of Δ98Δ (A, C) and Δ78Δ (B, D) in the absence (upper paneles) or in the presence of 10% TFE (lower panels). The right ordinate axes correspond to molecular weight data estimated by multi-angle static light scattering (MASLS). Average values (and standard deviations in Da) are the following: A: 12400 (1600); B: 16000 (900); C: 16700 (3700); D: 13700 (1600). (TIF) [file pone.0170607.s002.tif]

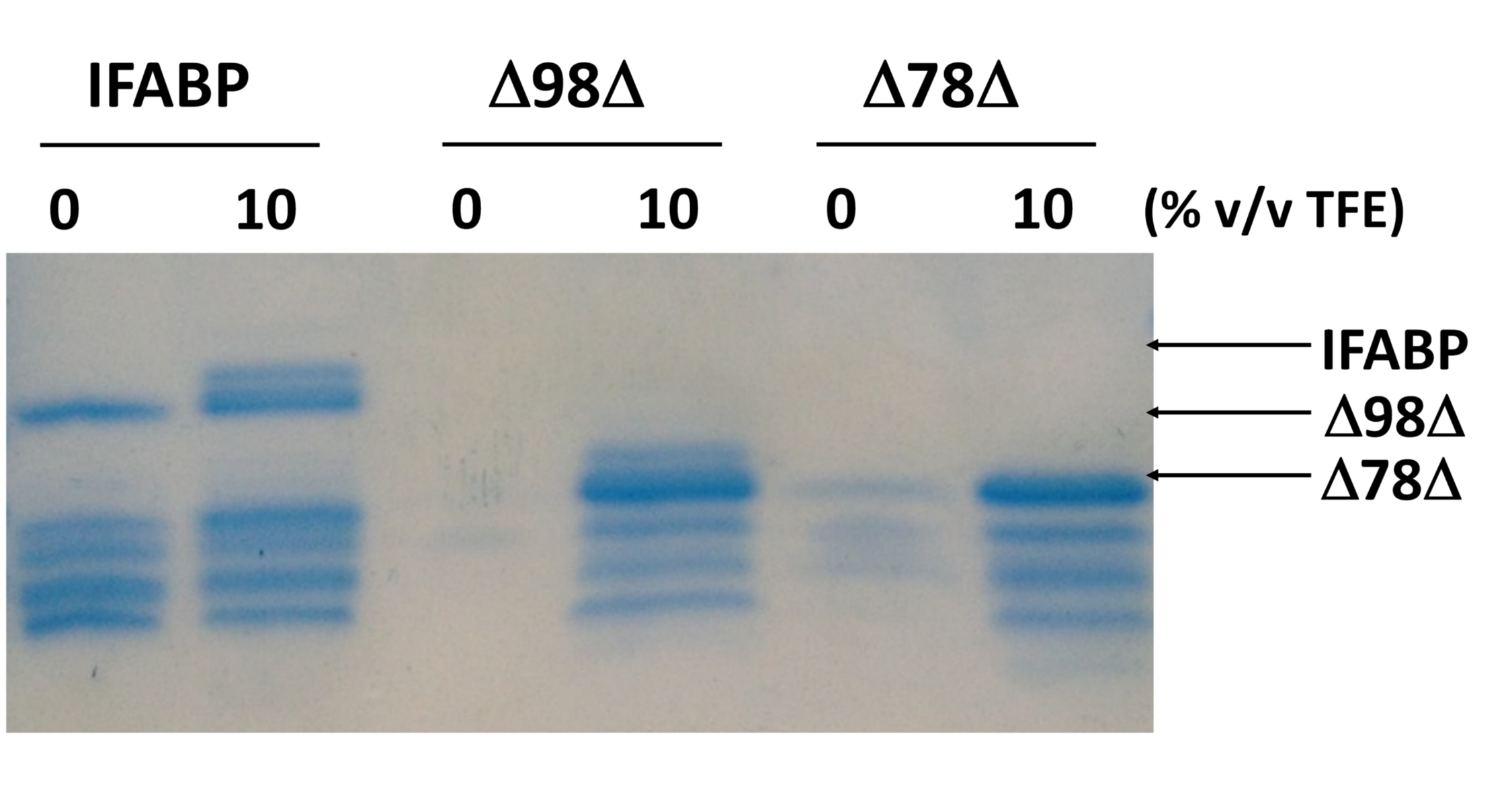

Supplement: S3 Fig — Separation by SDS-PAGE of the digestion mixture after treatment of proteins with proteinase K at 0 and 10% v/v TFE. Proteins were digested at a mass ratio of protein to protease of 200:1 for 30 min at 30°C. (TIF) [file pone.0170607.s003.tif]

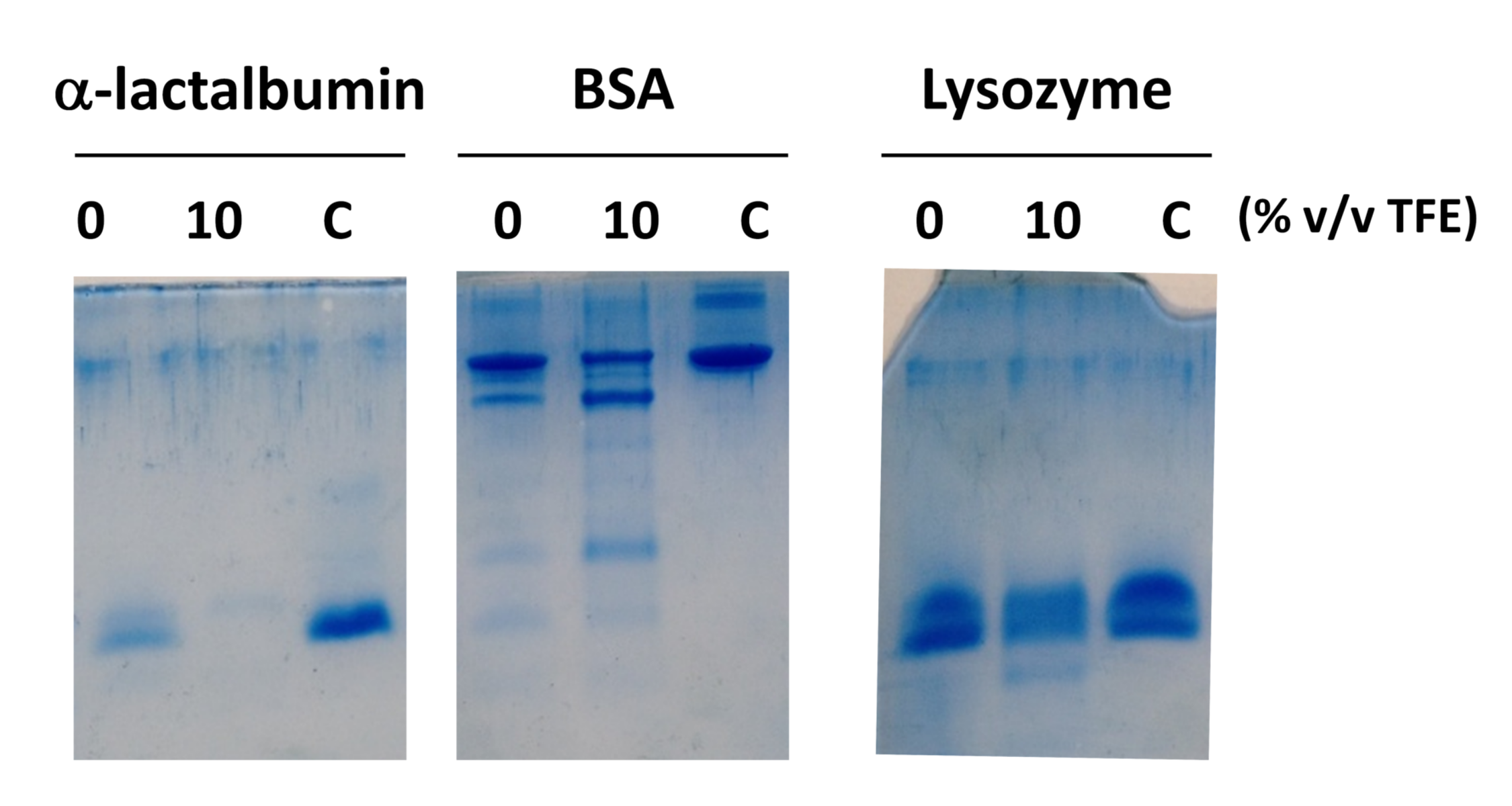

Supplement: S4 Fig — SDS-PAGE analysis of the fragments obtained by limited proteolysis with chymotrypsin at mass ratio of protein to protease of 10:1, overnight at 30°C. The lane labeled C corresponds to a control of protein load onto the gel. (TIF) [file pone.0170607.s004.tif]

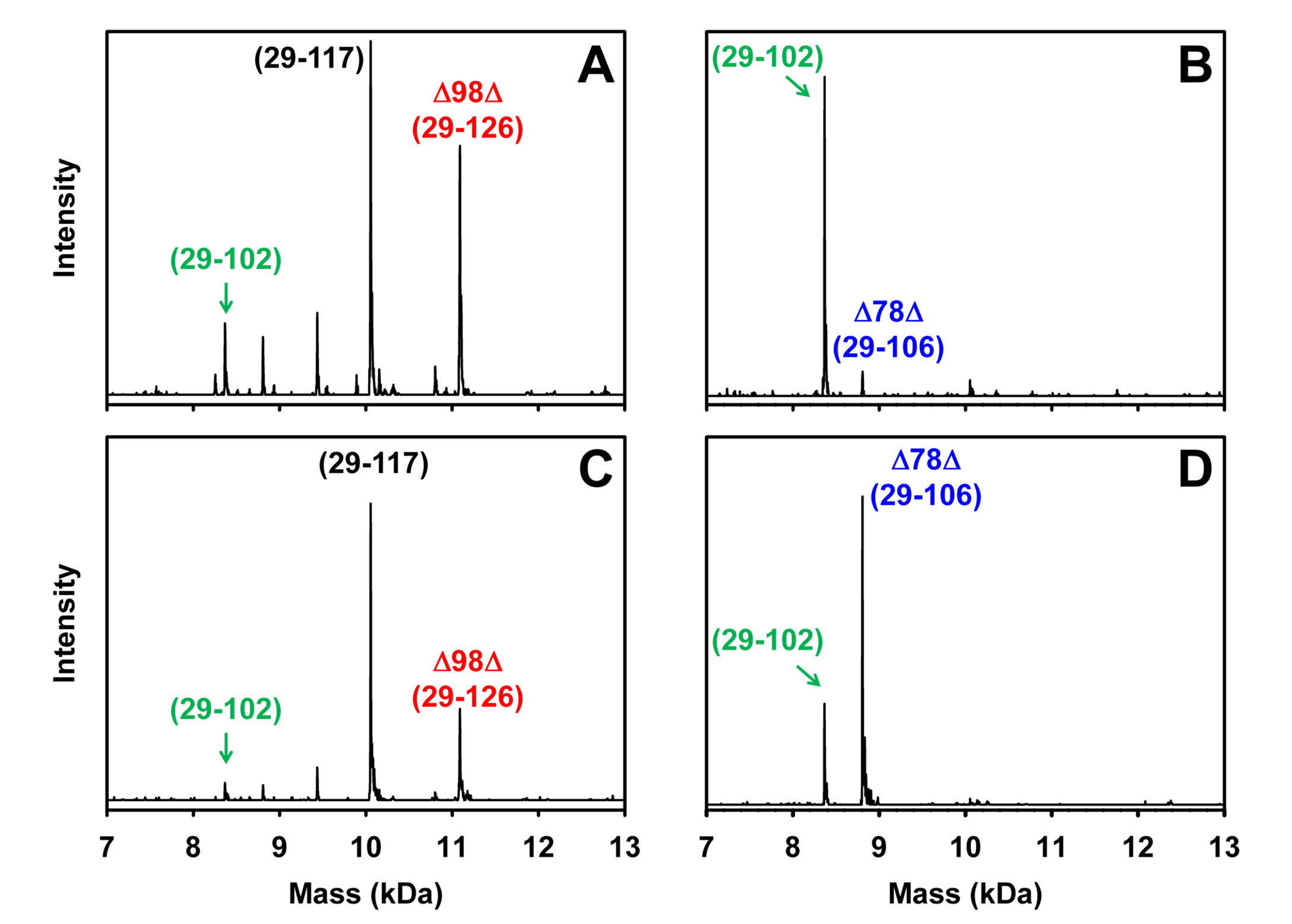

Supplement: S5 Fig — Proteins were digested at a mass ratio of protein to chymotrypsin of 200:1 at 30°C, either in the absence (upper paneles) or in the presence of 10% TFE (lower panels). The analysis of mixtures arising from Δ98Δ at 5 min (A) or 12 min (C) and those from Δ78Δ at 30 min (B and D) are shown in each panel. (TIF) [file pone.0170607.s005.tif]

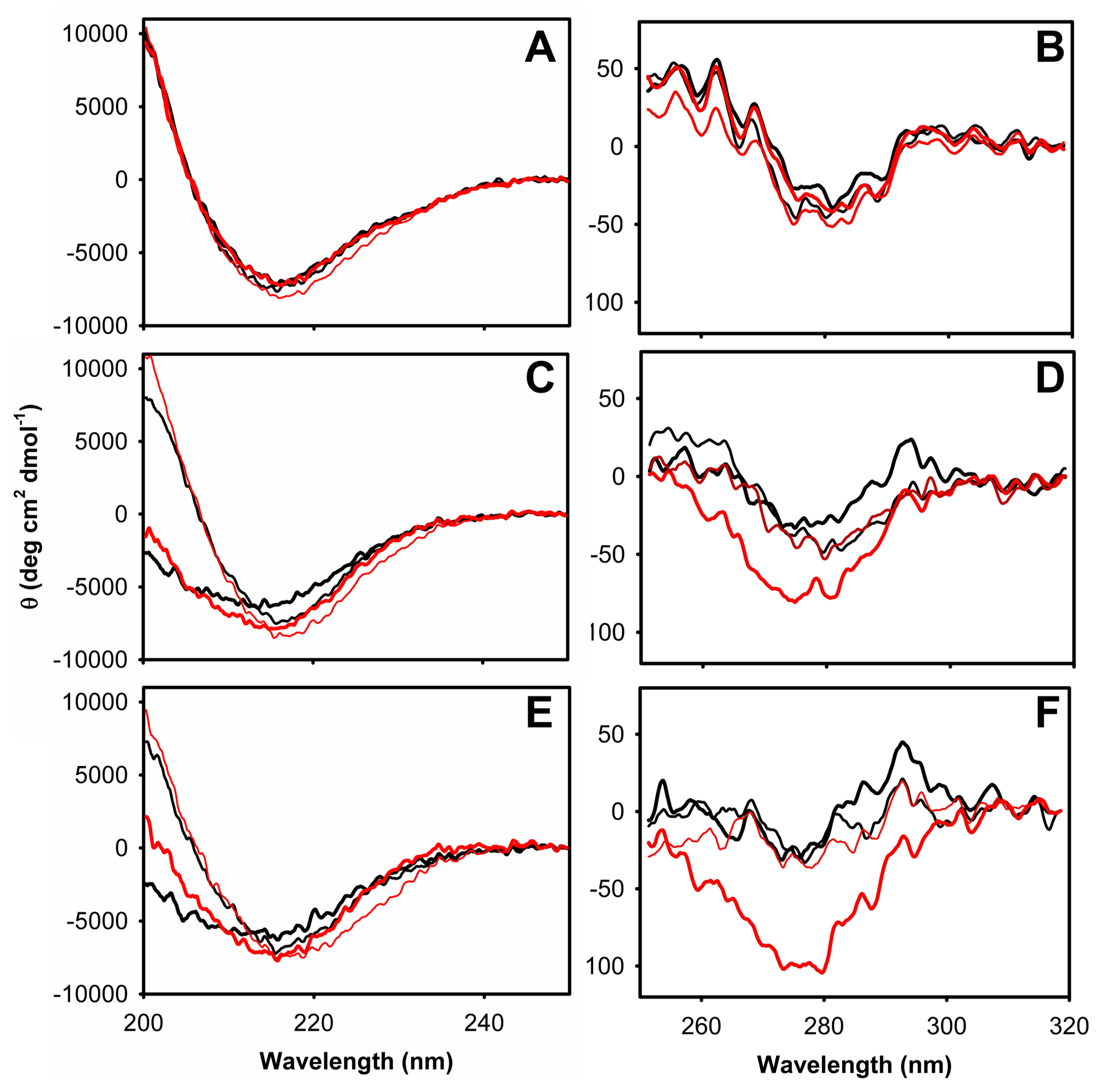

Supplement: S6 Fig — Oleic acid binding to IFABP (A and B), Δ98Δ (C and D) and Δ78Δ (E and F). Far (left panels) and near (right panels) UV CD spectra are shown at 0 (thick line) and 10 (thin line) % v/v TFE in buffer PN8 (see Materials and methods). Spectra recorded in the presence of oleic acid are represented in red lines. Smoothing of the traces (B, D and F) was achieved by averaging twice on a 15-point moving window (1.5 nm). The signature of oleic acid binding in the far UV CD region is a change of shape of the spectrum, characterized by a deepening and a trend toward incremental positive ellipticity at 200 nm (panels C and E). In the presence of 10% TFE, upon ligand binding a further deepening of the minimum and broadening in the range 215–235 nm are observed for all proteins. All in all, the effect of the co-solvent assimilates the response of the abridged variants to that observed for the parent protein. This evidence falls in place within a picture describing a general ordering effect on the truncated constructs. Consistently, the ligand-inducing effect appears as a significant enhancement of the magnitude of the near UV CD spectral signals observed for the abridged variants (panels D and F). (TIF) [file pone.0170607.s006.tif]

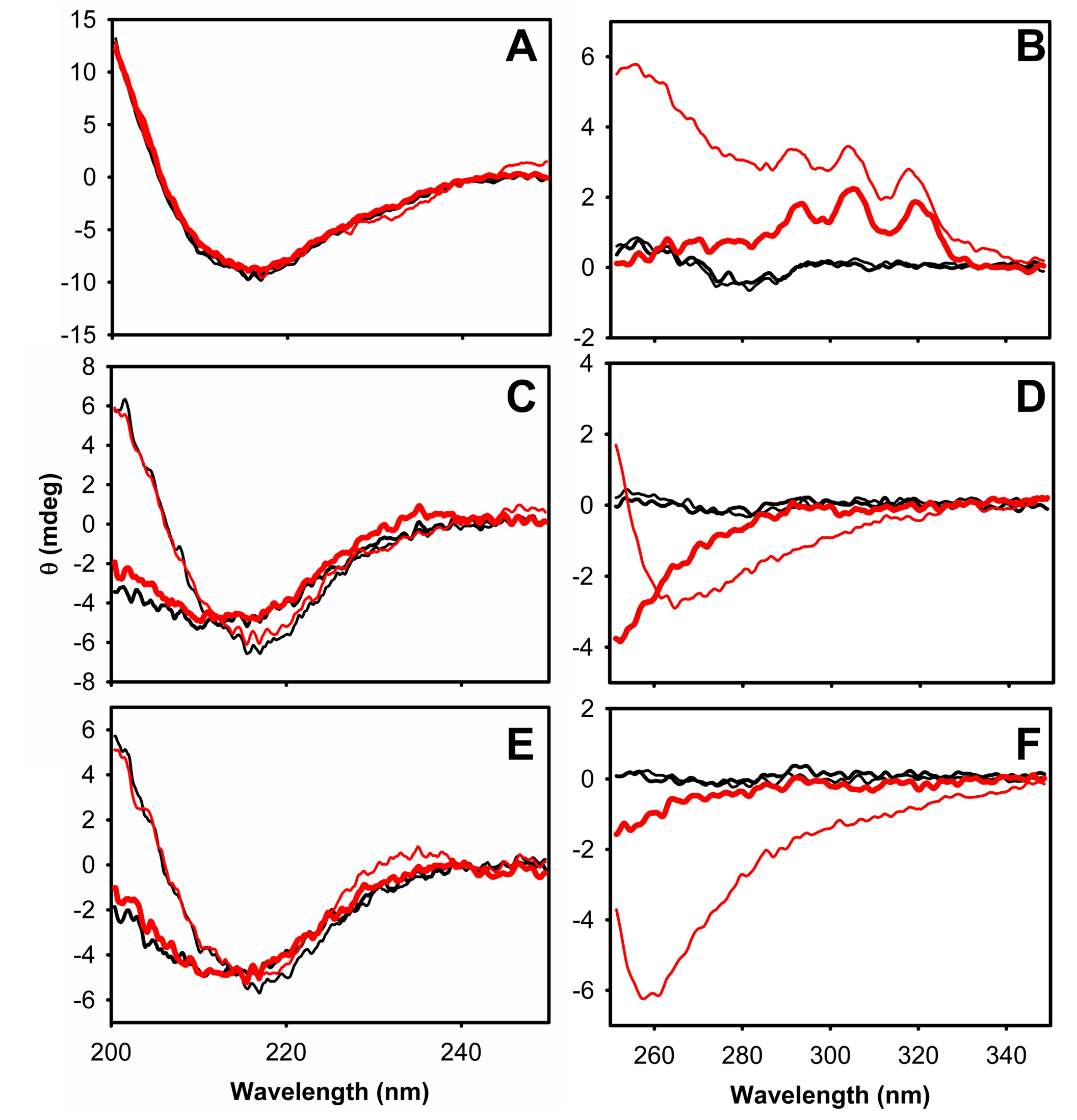

Supplement: S7 Fig — Binding of trans-parinaric acid (t-PA) to IFABP (A and B), Δ98Δ (C and D) and Δ78Δ (E and F). Far (left panels) and near (right panels) UV CD spectra are shown at 0 (thick line) and 10 (thin line) % v/v TFE in buffer PN8 (see Materials and methods). Spectra recorded in the presence of the fatty acid are represented in red lines. Smoothing of the traces (B, D and F) was achieved by averaging twice on a 15-point moving window (1.5 nm). For the abridged proteins in buffer, the new dichroic bands are: a negative one centered at ~ 247 nm and a positive band centered at ~ 235 nm (panels C-F). Notice the common iso-dichroic point at ~ 240 nm. For comparison, two very weak bands of opposite sign also appear in the case of IFABP. The more rigid framework provided by the wild-type protein places the ligand in a more asymmetric location, as evidenced by the appearance of a fine-structured CD spectrum in the near UV region (panels A and B). TFE leads to several perturbations in the spectra: (i) the intensity of the band centered at ~ 235 nm becomes higher for IFABP and Δ78Δ, whereas a red shift occurs for Δ98Δ; (ii) for all proteins, the band initially centered at ~ 247 nm suffers a dramatic red shift (~ 15 nm). For IFABP and Δ78Δ, a substantial enhancement of the intensity is also observed. (TIF) [file pone.0170607.s007.tif]

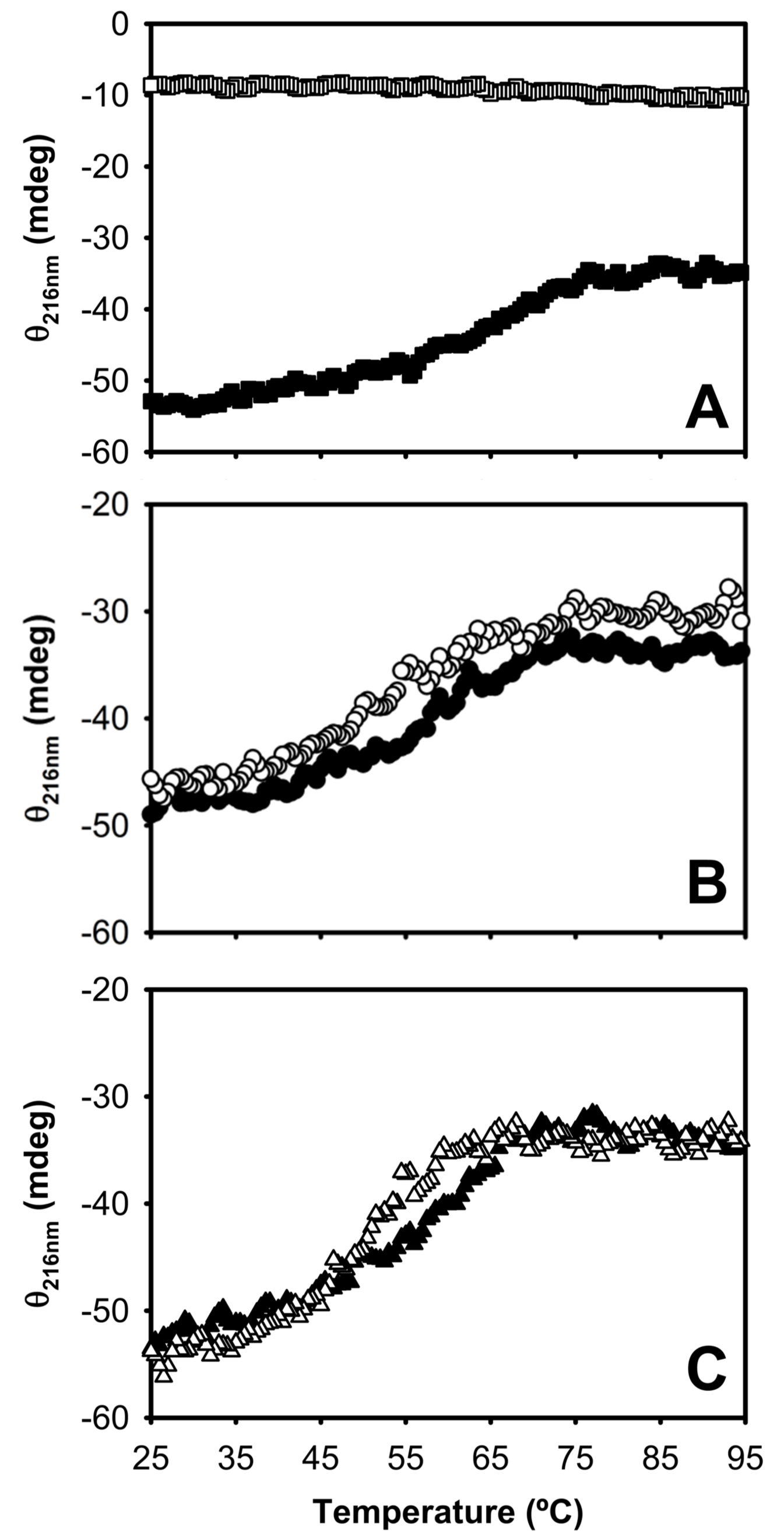

Supplement: S8 Fig — CD measurements recorded upon cooling samples of IFABP (A), Δ98Δ (B) and Δ78Δ (C) from 95 to 25°C. The temperature transitions were monitored by the evolution of the ellipticity at 216 nm at 0 (closed symbols), and 10 (open symbols) % v/v TFE. (TIF) [file pone.0170607.s008.tif]

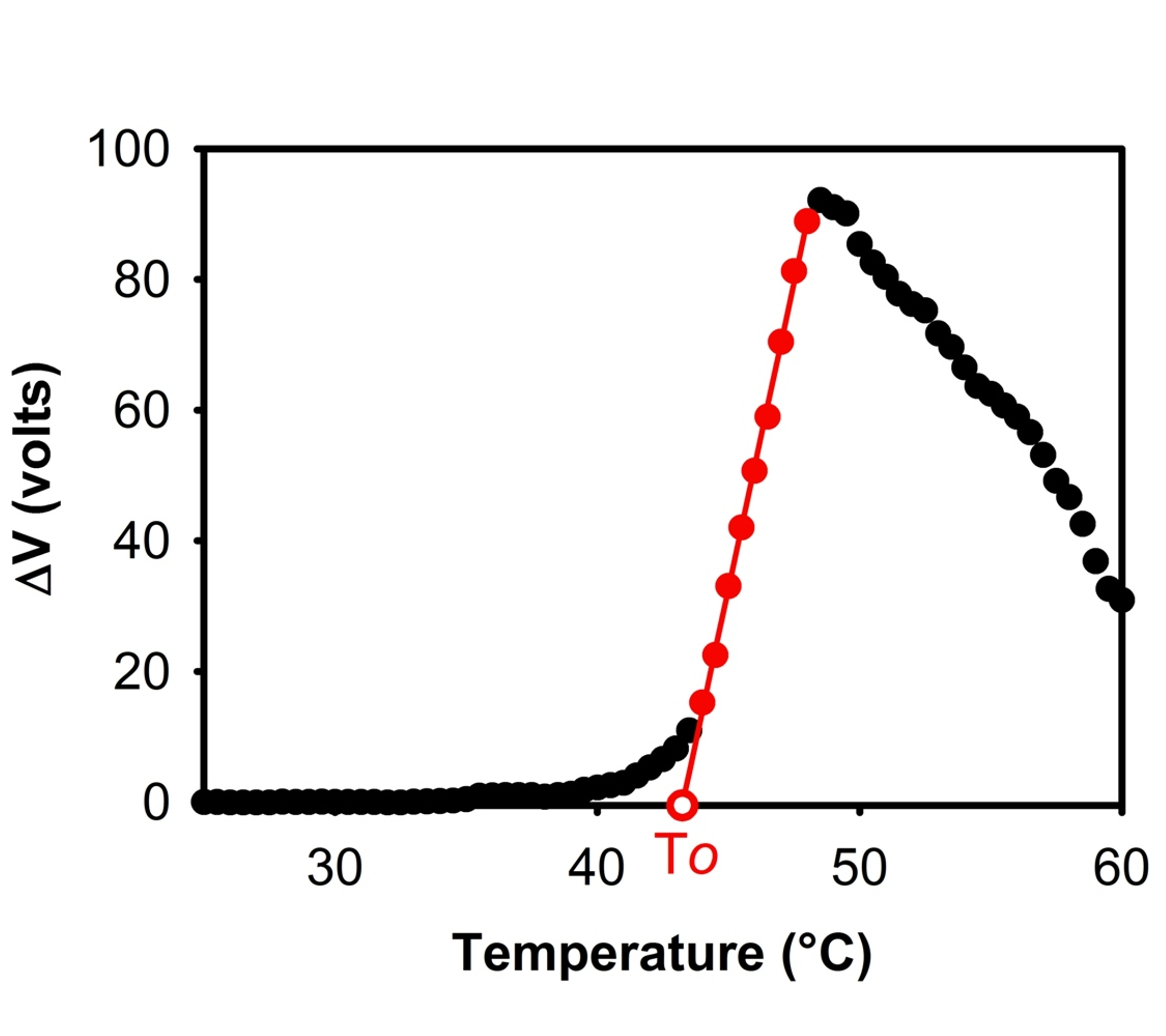

Supplement: S9 Fig — This value is derived after fitting a straight line to the initial time points, and extrapolating backwards to intersect the temperature abscissa. The monochromator was set at 216 nm. The voltage applied to the photomultiplier tube (PMT) of the spectropolarimeter is represented on the Y-axis, as the difference (ΔV) with respect to the value measured at 25°C. (TIF) [file pone.0170607.s009.tif]

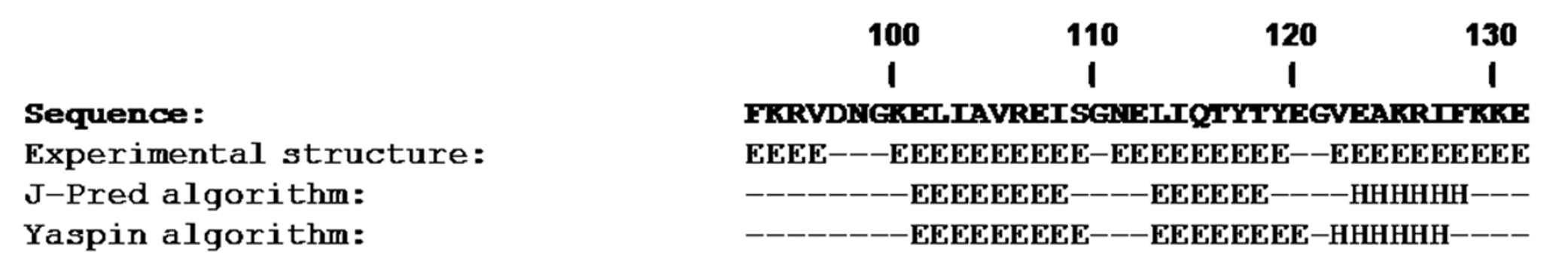

Supplement: S10 Fig — E and H stand for extended and helical conformations. Dashes correspond to positions where no defined secondary structure can be predicted or loops in the experimental structure. The latter was determined by X-ray crystallography (PDB 2IFB). (TIF) [file pone.0170607.s010.tif]
